# Predictors of Problematic Internet Use Among Romanian High School Students

**DOI:** 10.3390/children12101292

**Published:** 2025-09-24

**Authors:** Brigitte Osser, Csongor Toth, Carmen Delia Nistor-Cseppento, Mariana Cevei, Cristina Aur, Maria Orodan, Roland Fazakas, Laura Ioana Bondar

**Affiliations:** 1 Doctoral School of Biomedical Sciences, University of Oradea, 410087 Oradea, Romania; 2 Faculty of Physical Education and Sport, “Aurel Vlaicu” University of Arad, 310130 Arad, Romania; 3 Department of Psycho Neuroscience and Recovery, Faculty of Medicine and Pharmacy, University of Oradea, 410087 Oradea, Romania; cevei_mariana@uoradea.ro; 4 Department of Surgical Disciplines, Faculty of Medicine and Pharmacy, University of Oradea, 410073 Oradea, Romania; 5 Department of Biology and Life Sciences, Faculty of Medicine, “Vasile Goldiș” Western University of Arad, 310025 Arad, Romania; 6 Doctoral School of Medicine, “Vasile Goldiș” Western University of Arad, 310025 Arad, Romania

**Keywords:** adolescents, high school students, Internet Addiction Test, predictors, problematic internet use, screen time

## Abstract

**Highlights:**

What are the main findings?

- Male sex, older age, higher daily screen time, prior attempts to quit, and using the internet mainly for gaming were independent predictors of problematic internet use among Romanian adolescents.
- The model explained 43% of the variance in Internet Addiction Test (IAT) scores, highlighting distinct high-risk profiles.

What are the implications of the main findings?

- School-based screening and targeted interventions should prioritize older male students, heavy users, and those primarily engaged in gaming.
- Findings provide culturally specific evidence from Eastern Europe to inform adolescent digital-health policies and preventive programs.

**Abstract:**

Background: Problematic internet use among adolescents is linked to poorer mental health, academic performance, and social functioning, yet evidence from Eastern Europe remains limited. Methods: We conducted a school-based cross-sectional study at a Romanian high school (Arad County) including 308 students aged 15–18 years (154 males, 154 females). Students completed a demographic/behavioral questionnaire and the 20-item Internet Addiction Test (IAT), a widely used measure of problematic internet use. The prespecified primary analysis was a multivariable linear regression of IAT score on sex, age group, residence, daily screen time, prior attempts to reduce use, and main internet purpose; supporting analyses included *t*-tests, ANOVA, and Pearson correlation (α = 0.05). Results: In bivariable comparisons, males, older adolescents (17–18 years), and urban residents reported higher IAT scores; screen time correlated with IAT (r = 0.460, *p* < 0.001), and prior reduction attempts were associated with higher scores (Cohen’s d = 0.80). In the adjusted model, male sex (β = 4.97), older age (β = 5.36), greater daily screen time (β = 1.67 per hour), prior attempts to reduce use (β = 4.13), and primarily using the internet for gaming (β = 5.71) remained significant predictors (all *p* ≤ 0.045); urban residence was not retained (*p* = 0.218). The model explained 43% of IAT variance (R^2^ = 0.43). Conclusions: Demographic and behavioral factors independently predict adolescent problematic internet use, highlighting high-risk profiles (older males, heavy screen time, gaming focus, prior reduction attempts). These findings support school-based screening and targeted digital-health interventions in underrepresented contexts.

## 1. Introduction

Over the past two decades, digital technologies have become integral to adolescent life, shaping communication, education, and leisure activities. While the internet provides major benefits, excessive or maladaptive use—commonly referred to as problematic internet use—has been consistently associated with adverse outcomes such as impaired academic performance, reduced social relationships, and psychological distress [1,2,3,4]. Adolescents are especially vulnerable due to their ongoing cognitive and emotional development, increasing autonomy, and strong peer influences [5,6,7,8]. In this study, we use the term problematic internet use consistently while acknowledging that some prior literature also refers to it as internet addiction.

Prior research has highlighted a range of demographic and behavioral factors linked to problematic internet use. Male adolescents often display higher risk, largely driven by online gaming [9,10]. Older students report greater autonomy, higher academic stress, and longer daily screen time, all of which are associated with more severe internet use patterns [11,12,13]. Urban–rural differences have also been observed, reflecting unequal access to high-speed internet and differing cultural norms [13,14,15]. In addition, specific behavioral indicators—including the primary purpose of internet use (gaming, social media, or study), daily screen exposure, years of online experience, and previous attempts to reduce usage—are significant correlates of problematic use [16,17,18,19].

Despite substantial international research, data from Eastern Europe remain limited, leaving uncertainty about whether the findings from other contexts can be generalized. Romania represents a particularly relevant case: it has one of the fastest and most affordable internet infrastructures in Europe, yet digital education is inconsistently implemented. Furthermore, a large proportion of adolescents live in households where one or both parents work abroad, reducing parental supervision and potentially increasing reliance on digital media for social and emotional support. These socioeconomic and infrastructural conditions create a unique digital landscape that may amplify adolescents’ vulnerability to problematic internet use.

Adolescence is a critical stage when lifelong digital habits are formed. Excessive internet use has been linked to a range of health risks, including sleep problems, emotional difficulties, and heightened vulnerability to anxiety and depression [20,21]. Schools are a key setting for preventive action, as they can deliver digital literacy and early screening programs. Understanding local determinants of problematic internet use is therefore essential to guide interventions tailored to adolescents’ sociocultural realities [22,23,24].

At the same time, it is important to note that internet use is not inherently negative. Emerging evidence highlights that digital technologies, including mobile game-based educational interventions, can foster learning, metacognitive skills, motivation, and even environmental sensitivity in young learners. Recent reviews show that mobile games can enhance students’ metacognitive development and awareness of environmental factors, illustrating that the educational context and purpose of use play a crucial role in determining outcomes [25]. Acknowledging these benefits ensures a more balanced view, where risks are recognized but opportunities for positive digital engagement are also considered.

This study addresses this gap by investigating demographic and behavioral predictors of problematic internet use among Romanian high school students. Using Kimberly Young’s validated IAT, we examined how sex, age, residential background, screen time, primary online activity, and previous attempts to reduce use are associated with addiction severity. The balanced sample of 308 students allows for robust sex contrasts, while multivariable regression analysis identifies independent predictors, explaining 43% of the variance in IAT scores.

While an earlier study also applied the IAT to a Romanian high school sample, few such investigations have been conducted since [26]. By providing updated, culturally specific baseline evidence, our study contributes to the limited Eastern European literature and highlights actionable implications for school-based screening, digital literacy education, and adolescent mental-health policies.

## 2. Materials and Methods

### 2.1. Study Design and Setting

This research employed a cross-sectional design conducted at Vinga Technological High School, situated in Arad County, Romania. The study period spanned from April 2024 to April 2025, during which data was collected using a standardized, self-report questionnaire administered to students in classroom settings under the supervision of trained facilitators.

### 2.2. Study Population

A total of 308 high school students participated in the study. The sample included adolescents aged between 15 and 18 years, stratified into two developmental groups (15–16 and 17–18 years). Sex distribution was deliberately balanced (154 males and 154 females) to optimize the reliability of between-group comparisons and reduce sampling bias.

### 2.3. Inclusion and Exclusion Criteria

**Figure 1:**
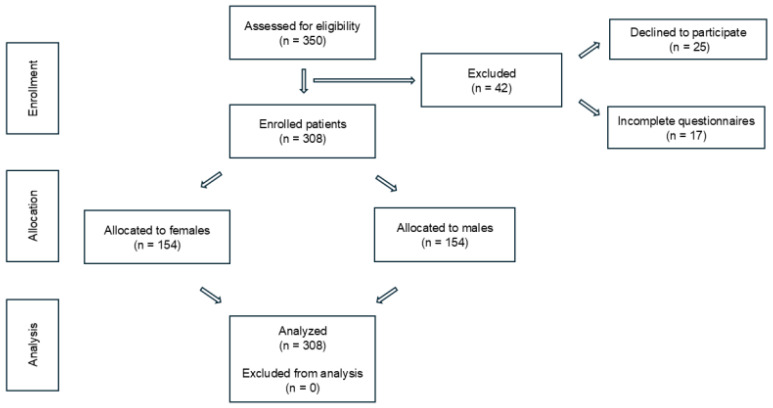
Flow diagram of participant enrollment, allocation, and analysis.

Participants were eligible if they were enrolled at Vinga Technological High School, aged 15–18 years and provided written informed consent. A total of 350 students were assessed; 42 were excluded—25 declined participation and 17 submitted incomplete questionnaires. The final sample included 308 students who met the criteria and completed the survey. Exclusion criteria included incomplete key sections of the questionnaire or cognitive impairments that might hinder independent understanding of survey items. A flow diagram summarizing the enrollment and analysis process is shown in Figure 1.

### 2.4. Data Collection Instruments

Data was collected through a structured instrument comprising several sections. Demographic variables included sex, age, and place of residence (urban or rural). Behavioral variables included average screen time, main purpose of internet use, years of internet experience, and whether students had previously attempted to reduce or quit internet usage. To assess the severity of problematic internet use, the IAT developed by Kimberly Young was employed. The IAT includes 20 items rated on a six-point Likert scale from 0 (“Does not apply”) to 5 (“Always”), yielding a total score ranging from 0 to 100, with higher scores indicating more severe problematic internet use. The IAT is a widely used and psychometrically validated instrument, with established reliability and construct validity in adolescent populations across diverse cultural contexts [27,28].

### 2.5. Ethical Considerations

Ethical approval for the study was obtained from the Ethical Committee of Vinga Technological High School (Approval No. 1436/2/16 October 2023). All participants were informed of the voluntary nature of the study and assured of the anonymity and confidentiality of their responses. Information assent was obtained from all student participants prior to participation. In addition, written informed consent was obtained from their parents or legal guardians. Both students and their guardians received an information sheet outlining the study’s purpose, procedures, data confidentiality, and the right to withdraw at any time without consequences.

### 2.6. Data Analysis

Data were analyzed using JASP 0.19.3 (University of Amsterdam, Amsterdam, The Netherlands) and Jamovi 2.3.28 (The Jamovi Project, Sydney, NSW, Australia). Descriptive statistics (means, standard deviations, frequencies, and percentages) were computed for all relevant variables.

Independent samples *t*-tests were conducted to compare IAT scores across sex, age groups, residence, and quit attempt history. A one-way analysis of variance (ANOVA) was performed to assess differences in IAT scores by primary internet use. Pearson correlation analysis was used to examine the relationship between daily screen time and IAT scores. Effect sizes were calculated for all relevant comparisons: Cohen’s d for *t*-tests and eta squared (η^2^) for ANOVA.

In addition, a multivariable linear regression analysis was conducted to identify independent predictors of IAT scores, including demographic and behavioral variables (sex, age, residence, screen time, quit attempts, and internet use purpose). Model fit was assessed using the coefficient of determination (R^2^). A *p*-value of less than 0.05 was considered statistically significant for all analyses.

Finally, internal consistency of the IAT was examined by computing Cronbach’s α (α = 0.89; 95% CI: 0.85–0.92).

### 2.7. Hypotheses of the Study

This study was guided by a series of hypotheses designed to explore the relationships between demographic characteristics, internet usage behaviors, and levels of problematic internet use among high school students. Drawing upon existing literature in adolescent psychology and digital behavior, the following hypotheses were formulated to guide the statistical analyses:

1. Male students will score significantly higher on the IAT than female students, consistent with prior findings that males are more likely to engage in excessive gaming and risk-taking online behaviors.
2. Older students (aged 17–18) will exhibit significantly higher IAT scores compared to younger students (aged 15–16), as older adolescents typically have more internet autonomy and academic demands.
3. Students residing in urban areas will have significantly higher IAT scores than those from rural areas, possibly due to greater access to fast internet and digital platforms in urban settings.
4. There will be a significant positive correlation between average daily screen time and IAT scores, aligning with prior studies linking high screen time to greater addiction risk.
5. A greater number of years of internet use will be associated with higher IAT scores, as longer exposure may normalize excessive use.
6. Students’ primary purpose of internet use will significantly influence their IAT scores, with social media and gaming use previously linked to higher addiction levels.
7. Students who have attempted to quit or reduce their internet use will report significantly higher IAT scores than those who have not, suggesting greater self-awareness of problematic usage patterns.

These hypotheses reflect the study’s aim to identify demographic and behavioral predictors of problematic internet use among adolescents and to evaluate the extent to which lifestyle and psychological factors contribute to risk. Each hypothesis was tested using appropriate statistical methods to ensure analytic rigor and consistency with existing empirical evidence.

## 3. Results

This section presents the main findings of the study. The results are organized according to relevant demographic characteristics and patterns of internet use. Descriptive statistics, group comparisons, and correlation analyses were conducted to explore potential differences and associations among the variables. Key outcomes are reported below, accompanied by tables and figures where appropriate.

### 3.1. Demographics

#### 3.1.1. Sex Differences in Problematic Internet Use

Male students reported higher IAT scores than female students, indicating that problematic internet use was more pronounced among boys (Table 1).

#### 3.1.2. Age Differences in Problematic Internet Use

Older students (17–18 years) reported higher IAT scores than younger students (15–16 years), indicating that problematic internet use tended to increase with age (Table 2).

#### 3.1.3. Residence Differences in Problematic Internet Use

Students from urban areas reported higher IAT scores than those from rural areas, suggesting a modest association between place of residence and problematic internet use (Table 3).

### 3.2. Digital Use Habits

#### 3.2.1. Correlation Between Screen Time and Problematic Internet Use

Daily screen time showed a moderate positive correlation with IAT scores, indicating that students who spent more hours online reported higher levels of problematic internet use (Table 4).

#### 3.2.2. Problematic Internet Use Scores by Quit Attempt History

Students who had tried to quit using the internet reported higher IAT scores than those who had not, suggesting greater addiction severity among students aware of problematic use (Table 5).

#### 3.2.3. Differences in Problematic Internet Use Based on Main Internet Use

The analysis revealed significant differences in IAT scores according to students’ primary internet activity. Those who mainly used the internet for social media or gaming had significantly higher scores than their peers who used it primarily for studying. No significant difference was observed between the social media and gaming groups. Streaming use fell between these categories but did not differ significantly from other groups (Table 6).

**Figure 2:**
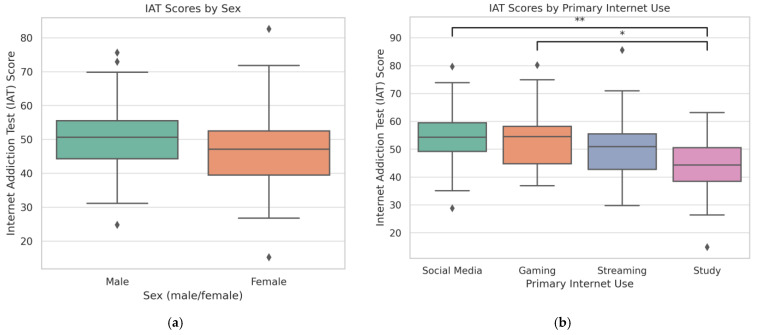
Distribution of IAT scores among Romanian high school students. (**a**) IAT scores by sex. Male students reported significantly higher scores than female students (*p* < 0.001). (**b**) IAT scores by primary internet use. Students who mainly used the internet for social media (** *p* < 0.01) or gaming (* *p* < 0.05) had significantly higher scores than those who used it for studying.

Figure 2 shows the distribution of IAT scores by sex and primary internet use. Male students displayed higher median scores and greater variability compared to females (panel a). Students who mainly used the internet for social media or gaming reported higher IAT scores than those who used it for studying or streaming (panel b).

### 3.3. Predictors of Problematic Internet Use

Regression analysis showed that being male, being 17–18 years old, higher daily screen time, prior attempts to quit, and using the internet mainly for gaming were significant independent predictors of higher IAT scores. Social media use showed a positive but borderline association, while residence was not significant (Table 7).

### 3.4. Reliability Analysis

IAT demonstrated excellent internal consistency in the present sample, with a Cronbach’s α of 0.89 (Table 8).

## 4. Discussion

This study found that male students, older adolescents, greater daily screen time, prior attempts to reduce internet use, and gaming as the main online activity were independent predictors of problematic internet use, together explaining 43% of the variance in IAT scores. To our knowledge, this is among the first school-based studies in Romania applying the IAT, providing baseline data for Romanian adolescents and contributing to the limited evidence available from Eastern Europe. Importantly, the IAT demonstrated excellent internal consistency in this sample (α = 0.89, 95% CI: 0.85–0.92), consistent with reliability values reported in adolescent populations across diverse cultural contexts [29,30].

These findings are especially relevant given Romania’s unique digital environment, marked by very high internet penetration, limited parental supervision in many households due to labor migration, and uneven implementation of digital education policies. Our results also align with global evidence in several ways. Consistent with international studies, males were at greater risk, largely due to gaming behaviors, while older adolescents showed higher vulnerability linked to greater autonomy and academic stress. The apparent urban–rural difference disappeared in the adjusted model, suggesting that online behaviors, not residence, drive risk. Together, these results emphasize the need for region-specific prevention programs in schools, particularly targeting high-risk groups such as male students, older adolescents, and heavy gamers.

### 4.1. Sex Differences

The findings of this study indicate that male students scored significantly higher on the IAT compared to female students, with a medium effect size (d = 0.50). This result is consistent with previous research showing that adolescent males are more vulnerable to problematic internet use, especially in contexts involving online gaming and recreational browsing. Males often report more time spent on immersive digital platforms that are highly stimulating and socially competitive—such as multiplayer video games or streaming services—compared to females, who typically engage more in communication and educational uses of the internet [2,16,31].

This sex disparity may be rooted in both behavioral tendencies and socialization patterns. Male adolescents are often encouraged to explore mastery, autonomy, and achievement in online spaces, which can lead to prolonged engagement and potential dependence [10]. In contrast, female students tend to use the internet more relationally—for communication, social media, or academic collaboration—which are typically less associated with compulsive usage [4]. Moreover, some studies suggest that impulsivity and reward sensitivity, more commonly reported in male users, may contribute to a stronger susceptibility problematic or addictive online behaviors [32]. These results suggest that sex-sensitive interventions are warranted, particularly those aimed at helping male students develop balanced digital habits and awareness of screen time limits [2].

### 4.2. Age-Related Trends

The results indicated that older students (aged 17–18) exhibited significantly higher scores on the IAT compared to younger students (aged 15–16), with a medium effect size (d = 0.52). This finding is consistent with previous studies that have shown a steady increase in problematic internet use during late adolescence. As adolescents mature, their autonomy and access to technology typically increase, granting them greater control over their online behavior. At the same time, their digital habits may intensify as they balance academic demands, social relationships, and emotional development—factors that can elevate vulnerability to compulsive or problematic internet engagement [12,33,34,35].

Older teens may also use the internet more frequently as a means of managing stress, avoiding offline responsibilities, or maintaining social connections, particularly through social media and entertainment platforms. These patterns are reinforced by the greater academic pressure and decision-making burden that often accompany the final years of secondary education. Additionally, neurological and psychological changes during late adolescence, including heightened impulsivity and sensation seeking, may further contribute to prolonged screen time and increased risk for problematic internet use and related maladaptive behaviors. These results support the need for age-appropriate digital health education and coping strategies, particularly for upper-year students navigating academic and social transitions [12,36].

### 4.3. Urban–Rural Disparities

The analysis revealed that students from urban areas reported significantly higher IAT scores compared to their rural peers, with a small-to-medium effect size (d = 0.43). This finding is in line with national and international research suggesting that problematic internet use is more prevalent in urban regions, where technological infrastructure, digital device ownership, and broadband coverage are more advanced [15,37]. Urban adolescents often have greater access to smartphones, Wi-Fi, and high-speed networks, which not only facilitates frequent internet use but also increases the likelihood of habitual or compulsive behaviors [14,38,39].

Beyond infrastructure, environmental and cultural factors may also play a role. Urban environments tend to promote faster-paced lifestyles, greater exposure to social media trends, and increased use of technology in both educational and recreational settings. These conditions may encourage adolescents to rely more heavily on digital platforms for communication, entertainment, and even emotional regulation. In contrast, rural youth may experience fewer digital stimuli, more outdoor or face-to-face interactions, and cultural norms that prioritize offline engagement. These findings underscore the importance of considering regional and contextual factors in developing digital literacy programs and preventive interventions tailored to different geographic populations [38,39,40].

However, in the multivariable regression model, urban residence was no longer a significant predictor of problematic internet use, suggesting that the observed differences are explained by behavioral variables such as screen time and type of online activity. In other words, it is not where adolescents live but how they use the internet that matters. This highlights the importance of targeting specific digital practices, such as prolonged gaming or social media engagement, rather than focusing interventions on geography.

### 4.4. Screen Time and Quit Attempts

The study identified a moderate positive correlation between daily screen time and IAT scores (r = 0.460), accounting for approximately 21% of the variance. This finding aligns with a substantial body of research indicating that prolonged screen exposure is a key risk factor for the development of problematic or compulsive internet use behaviors. Excessive screen time, particularly when unstructured or unmonitored, can displace essential activities such as physical exercise, in-person social interaction, and academic focus—further reinforcing digital dependency. In adolescence, when self-regulation is still developing, increased exposure to interactive and stimulating content—like social media feeds, video games, or streaming platforms—may significantly heighten the risk of problematic internet use [4,41,42].

Additionally, students who reported previous attempts to quit or reduce their internet use had significantly higher IAT scores than those who had not, with a large effect size (d = 0.80). This indicates that more severe users may recognize their problematic behavior but struggle to sustain change. In line with behavioral addiction theories, awareness often triggers attempts to cut back, yet relapse is common due to strong reward loops, inadequate coping resources, and reliance on the internet for stress relief or social connection. In the Romanian context, where many adolescents experience limited parental supervision due to labor migration, these self-regulation attempts often occur without adequate guidance or external support, which may explain why they fail. Practically, a history of quit attempts could serve as a useful screening indicator in schools and clinical settings, helping to identify adolescents at highest risk who would benefit most from structured interventions, digital detox programs, and psychological support [43,44,45,46,47].

### 4.5. Primary Internet Use and Problematic Internet Use

The analysis revealed a statistically significant difference in IAT scores based on students’ primary use of the internet. Those who primarily engaged in social media or gaming reported markedly higher levels of problematic internet use compared to peers who used the internet mainly for academic purposes. This pattern is consistent with previous literature suggesting that interactive and highly stimulating online activities—such as social networking and video gaming—are more strongly associated with problematic or addictive behaviors. These platforms often incorporate reward systems, social feedback loops, and immersive content that encourage prolonged and repeated use, which may disrupt daily routines and contribute to compulsive digital engagement [48,49,50]. In gaming, achievement systems, competition, and multiplayer interaction create powerful reinforcement cycles, while social media platforms exploit mechanisms such as social feedback, fear of missing out, and constant comparison. These design features encourage repetitive engagement and can make withdrawal difficult, particularly in adolescents with heightened reward sensitivity.

In contrast, students who reported studying or streaming as their main internet activity exhibited lower IAT scores, reflecting more structured and goal-directed forms of use. Passive content consumption and academic engagement typically lack the immediate reinforcement and high-arousal features present in gaming or social networking environments. This distinction underscores that not all screen time carries equal risk: the purpose and psychological design of online activities are critical in shaping addictive potential [51,52,53].

These findings indicate that prevention programs should move beyond simple screen time reduction to focus on the quality of digital engagement. Educational policies and wellness initiatives should address the addictive design of gaming and social media, while simultaneously promoting constructive online activities—such as academic, creative, or informational uses—that provide benefits without reinforcing compulsive patterns.

### 4.6. Implications and Context

Taken together, the findings of this study suggest that adolescent problematic internet use is shaped by a combination of sociodemographic and behavioral factors rather than being a uniform or random phenomenon. Variations in problematic use by sex, age, and purpose of internet use highlight the importance of a multifaceted approach in both research and intervention [54,55,56]. The elevated scores among male students, older adolescents, and heavy gamers emphasize the need for contextualized preventive strategies that reflect the digital realities specific to these groups. Moreover, the strong association between higher IAT scores and attempts to quit suggests that many students are aware of their problematic use but may lack the necessary tools or support to make lasting changes [57,58].

These insights carry practical implications for educators, mental health professionals, and policymakers aiming to promote digital well-being in youth populations. Digital literacy programs should be embedded early in the school curriculum and adapted to account for diverse usage patterns and access levels [59,60]. Importantly, not all online activities contribute equally to problematic use risk; therefore, public health messaging and school-based interventions should distinguish between high-risk interactive behaviors (e.g., gaming, social media) and more functional or passive use (e.g., studying, streaming). By promoting healthy, goal-oriented engagement with technology and addressing the psychosocial contexts that drive excessive use, schools and communities can help mitigate the growing challenge of problematic internet use among adolescents [61,62,63].

Finally, the identification of sex, age group, daily screen time, quit attempts, and internet use purpose as independent predictors underscores the value of multivariable approaches in detecting high-risk student profiles. Such models can inform more focused prevention strategies, ensuring that interventions are not only evidence-based but also tailored to the Romanian and broader Eastern European context.

Overall, these findings provide one of the first evidence-based profiles of Romanian adolescents at risk for problematic internet use, underscoring the urgency of developing targeted prevention strategies in schools and communities. Although our findings focus on predictors of problematic internet use, prior research emphasizes that digital technologies can also serve adaptive and educational functions. For example, mobile game-based interventions have been shown to support young learners’ metacognitive development, motivation, and environmental sensitivity [25]. This suggests that preventive strategies should not pathologize all internet use, but rather distinguish between harmful patterns and constructive, goal-oriented engagement. Promoting balanced digital habits may therefore involve both reducing risky behaviors and fostering beneficial applications of technology within educational contexts.

### 4.7. Limitations and Future Directions

This study has several limitations that should be considered when interpreting the findings. First, the cross-sectional design prevents conclusions about causal relationships between internet use patterns and problematic behaviors. It remains unclear whether high screen time leads to problematic tendencies, or whether adolescents with higher vulnerability are more likely to engage in excessive internet use. Second, the reliance on self-reported questionnaires may have introduced bias, including inaccurate recall or socially desirable responses, especially regarding sensitive behaviors such as excessive screen time or attempts to quit. Third, the study was conducted in a single technological high school in Arad County with a relatively modest sample size. Although the sex distribution was balanced, the restricted setting and regional focus limit the generalizability of results to other schools, age groups, or cultural contexts. Finally, the study did not account for certain psychological and contextual factors such as impulsivity, anxiety, self-esteem, or family dynamics that may also shape adolescents’ risk for problematic internet use.

Future research should address these limitations by adopting longitudinal designs that can clarify developmental trajectories and causal pathways. Including psychological and family-related variables would provide a more comprehensive understanding of the mechanisms underlying problematic internet use. Moreover, multi-site or nationally representative studies are needed to strengthen external validity and capture regional differences. Complementary qualitative methods, such as interviews or focus groups, may also reveal adolescents’ personal and social motivations for excessive internet use, adding depth to quantitative findings. Together, such approaches could inform more nuanced and context-sensitive intervention strategies.

## 5. Conclusions

This study highlights the multifactorial nature of problematic internet use among adolescents, demonstrating significant differences based on sex, age, residential background, screen time habits, and primary purpose of internet use. Male students, older adolescents, and those living in urban areas were found to be at higher risk, while activities such as social media and gaming were more strongly associated with problematic usage than academic or passive online behavior. These findings were supported by multivariable regression analysis, which identified sex, age, screen time, quit attempts, and internet use purpose as independent predictors of IAT scores, accounting for approximately 43% of the variance.

Given the increasing integration of digital media into educational and social domains, it is essential to promote balanced and informed technology use among adolescents. Interventions should be tailored to demographic risk profiles and usage patterns, focusing not just on reducing screen time but on fostering healthy digital habits and emotional coping strategies. These insights can support the development of targeted prevention strategies within schools and communities.

Finally, by providing statistically robust and culturally relevant data from an underrepresented Eastern European population, this study contributes to the global literature on adolescent problematic internet use. These findings should inform national adolescent health strategies and educational reforms aimed at mitigating the risks of digital overuse and promoting digital well-being in diverse youth populations.

## Figures and Tables

**Table 1:** Sex Differences in IAT Scores.

Group	N	M	SD	t	df	*p*	d
Male	154	51.4	10.2	3.82	306	<0.001	0.50
Female	154	46.2	9.5	—	—	—	—

Note: M = Mean; SD = Standard Deviation; t, df, *p* = results of the independent samples *t*-test. d = Cohen’s d, measuring effect size.

**Table 2:** Age Group Differences in IAT Scores.

Group	N	M	SD	t	df	*p*	d
17–18 years	158	52.4	9.7	3.72	306	<0.001	0.52
15–16 years	150	47.3	10.2	—	—	—	—

Note: M = Mean; SD = Standard Deviation; t, df, and *p* = results of the independent samples *t*-test. d = Cohen’s d, measuring effect size.

**Table 3:** Urban–Rural Differences in IAT Scores.

Group	N	M	SD	t	df	*p*	d
Urban	185	51.8	9.9	3.17	306	0.002	0.43
Rural	123	47.6	10.5	—	—	—	—

Note: M = Mean; SD = Standard Deviation; t, df, and *p* = results of the independent samples *t*-test. d = Cohen’s d, measuring effect size.

**Table 4:** Correlation Between Screen Time and IAT Scores.

Variable		IAT Score	Screen Time
IAT Score	Pearson’s r	—	
	*p*-value	—	
Screen Time	Pearson’s r	0.460	—
	*p*-value	<0.001	—

Note: Pearson correlation was used to assess the relationship between total IAT scores and average daily screen time (in hours). r^2^ = 0.21.

**Table 5:** Differences in IAT Scores Based on Attempt to Quit Internet Use.

Group	N	M	SD	t	df	*p*	d
Tried to quit	108	55.1	9.5	5.84	306	<0.001	0.80
Not tried	200	47.2	10.3	—	—	—	—

Note: M = Mean; SD = Standard Deviation; t, df, and *p* = independent samples *t*-test. d = Cohen’s d, measuring effect size.

**Table 6:** IAT Scores by Main Internet Use Purpose with ANOVA and Post Hoc Test Results.

Main Use	N	M	SD	Significant Post Hoc Comparisons (Tukey)
Social media	123	55.2	9.8	>Studying (*p* < 0.01)
Gaming	77	53.1	10.0	>Studying (*p* < 0.01)
Streaming	46	48.6	9.5	n.s.
Studying	62	44.3	8.9	Reference group

Note: One-way ANOVA showed a statistically significant difference in IAT scores by main internet use, F (3, 304) = 14.6, *p* < 0.001, η^2^ = 0.13. Tukey post hoc tests confirmed that the social media and gaming groups scored significantly higher than the studying group. n.s. = not significant.

**Table 7:** Multivariable Linear Regression Predicting IAT Scores.

Predictor Variable	Coefficient (β)	Std. Error	t-Value	*p*-Value
Constant	48.53	6.77	7.17	<0.001
Sex (male)	4.97	2.26	2.20	0.045
Age Group (17–18)	5.36	2.19	2.44	0.026
Residence (urban)	2.61	2.02	1.29	0.218
Daily Screen Time	1.67	0.68	2.45	0.025
Attempted to Quit (yes)	4.13	1.86	2.21	0.044
Main Use: Gaming	5.71	2.56	2.23	0.042
Main Use: Social Media	4.85	2.40	2.02	0.058

Reference categories are female (sex), age 15–16, rural residence, and studying (internet use purpose).

**Table 8:** Overall Scale Reliability Statistics.

Estimate	Cronbach’s α
Point estimate	0.89
95% CI lower bound	0.85
95% CI upper bound	0.92

## Data Availability

The data presented in this study are available on reasonable request from the corresponding author. The data are not publicly available due to privacy and ethical restrictions related to the adolescent participants.

## Abbreviations

The following abbreviation is used in this manuscript:

IAT	Internet Addiction Test
